# Melatonin, BAG-1 and cortisol circadian interactions in tumor pathogenesis and patterned immune responses

**DOI:** 10.37349/etat.2023.00176

**Published:** 2023-10-25

**Authors:** George Anderson

**Affiliations:** Sun Yat-Sen University Cancer Center, China; CRC Scotland & London, Eccleston Square, SW1V1PG London, UK

**Keywords:** Tumor pathogenesis, melatonin, *N*-acetylserotonin, aryl hydrocarbon receptor, cortisol awakening response, hypothalamus-pituitary-adrenal axis, B cell lymphoma-2-associated athanogene 1, mitochondria

## Abstract

A dysregulated circadian rhythm is significantly associated with cancer risk, as is aging. Both aging and circadian dysregulation show suppressed pineal melatonin, which is indicated in many studies to be linked to cancer risk and progression. Another independently investigated aspect of the circadian rhythm is the cortisol awakening response (CAR), which is linked to stress-associated hypothalamus-pituitary-adrenal (HPA) axis activation. CAR and HPA axis activity are primarily mediated via activation of the glucocorticoid receptor (GR), which drives patterned gene expression via binding to the promotors of glucocorticoid response element (GRE)-expressing genes. Recent data shows that the GR can be prevented from nuclear translocation by the B cell lymphoma-2 (Bcl-2)-associated athanogene 1 (BAG-1), which translocates the GR to mitochondria, where it can have diverse effects. Melatonin also suppresses GR nuclear translocation by maintaining the GR in a complex with heat shock protein 90 (Hsp90). Melatonin, directly and/or epigenetically, can upregulate BAG-1, suggesting that the dramatic 10-fold decrease in pineal melatonin from adolescence to the ninth decade of life will attenuate the capacity of night-time melatonin to modulate the effects of the early morning CAR. The interactions of pineal melatonin/BAG-1/Hsp90 with the CAR are proposed to underpin how aging and circadian dysregulation are associated with cancer risk. This may be mediated via differential effects of melatonin/BAG-1/Hsp90/GR in different cells of microenvironments across the body, from which tumors emerge. This provides a model of cancer pathogenesis that better integrates previously disparate bodies of data, including how immune cells are regulated by cancer cells in the tumor microenvironment, at least partly via the cancer cell regulation of the tryptophan-melatonin pathway. This has a number of future research and treatment implications.

## Introduction

There is a growing interest in role of mitochondrial function in determining core aspects of intracellular and intercellular interactions across diverse medical conditions, including autoimmune/immune-mediated disorders [1, 2] and neurodegenerative/neuropsychiatric disorders [3–5]. The importance of mitochondrial function and alterations in tumor cellular metabolism have been appreciated since the time of Warburg to be an important determinant of cancer pathogenesis and pathophysiology [6]. Recent work indicates that core aspects of mitochondrial function are also important determinants of the dynamic changes occurring in other cells of the tumor microenvironment, with tumor cells having a major role in the modulation of mitochondrial function in other tumor microenvironment cell types [7, 8]. As mitochondria are the major cellular source of reactive oxygen species (ROS), which drives ROS-mediated microRNAs (miRNAs) that shape patterned gene expression [9], intercellular “metabolic interactions” are significant contributors to the dynamic changes in intercellular fluxes among cells in the tumor microenvironment [8].

Sleep and circadian dysregulation are important risk factors for many cancers, including breast cancer [10] and lung cancer [11]. Preclinical data shows chronic sleep disruption to suppress the antitumor immune response [12], as well as to modulate tumor progression in breast cancer patients [13]. The circadian disruption in breast cancer patients includes suppression of the night-time melatonin production by the pineal gland and alterations in the cortisol awakening response (CAR) [13, 14]. Alterations in the circadian rhythm are also strongly associated with mood, fatigue, and cognition as well as with treatment outcomes [15]. Much of the work on the role of the circadian rhythm in cancer pathogenesis and pathophysiology has focused on changes in pineal melatonin [16] and CAR [17] as distinct circadian processes.

Melatonin is widely appreciated to have a significant role in cancer treatment, including via the induction of apoptosis in tumors [18], and decreasing metastasis [19] as well as attenuating chemotherapy [20] and radiotherapy side-effects [21]. Although primarily known for its release by the pineal gland at night, the melatonergic pathway is evident in every body cell investigated to date, primarily within mitochondria [22]. The regulation of the tryptophan-melatonin pathway is increasingly recognized as an important aspect of intercellular interactions in a given microenvironment, being a significant target as to how one cell influences and modulates the function of a neighboring cell [2]. Variations in the regulation of the mitochondrial melatonergic pathway are also an important determinant of cell phenotypic and plasticity responses and therefore of the dynamic interactions occurring in a given microenvironment, including within the immune cells and other cells of the tumor microenvironment [8]. This is exemplified in the two immune cells where the mitochondrial melatonergic pathway has been most extensively investigated, namely macrophages [23] and microglia [24], where the shift from an M1-like pro-inflammatory phenotype to an M2-like pro-phagocytic phenotype is mediated by autocrine melatonin [23, 24]. Variations in pineal melatonin therefore have important implications for the regulation of immune responses, including via metabolic and mitochondrial function.

The article highlights how the pathogenesis of the tumor microenvironment can be conceptualized as driven by dynamic, intercellular mitochondrial interactions, of which the mitochondrial melatonergic pathway is a core aspect. It is proposed that circadian processes, namely pineal melatonin and the CAR interact via the capacity of melatonin to upregulate B cell lymphoma-2 (Bcl-2)-associated athanogene 1 (BAG-1), which drives the translocation of the glucocorticoid receptor (GR) to mitochondria and away from the nucleus, thereby having significant impacts on mitochondrial function and patterned gene expression. Factors that suppress melatonin, such as aging and inflammation, will therefore have consequences as to how CAR acts to prime body cells, including immune cells, with differential effects in different cell types, thereby altering the homeostatic interactions within a given microenvironment. The article integrates wider previously disparate bodies of data on cancer pathogenesis and tumor microenvironment intercellular interactions, with a number of future research and treatment implications. First, the tryptophan-melatonin pathway is briefly described.

## Tryptophan-melatonin pathway

The tryptophan-melatonin pathway is present in all human (and multicellular organism) cells so far investigated [25]. This is proposed to arise from the common ancestor of multicellular life, an ancient bacterium that crept into a single cell organism, being a melatonin producing bacteria [25]. Over the course of 2 billion years of evolution, all species of the three kingdoms of multicellular life on Earth (animals, plants, and fungi) have retained the capacity to produce melatonin, typically within mitochondria [25]. Such maintenance of the mitochondrial melatonergic pathway by multi-cellular life over 2 billion years of evolutionary challenges highlight the importance of melatonin to multicellular life on Earth.

The melatonergic pathway is initiated by dietary- and shikimate pathway-derived tryptophan uptake, which enters the circulation where it is taken into cells by large amino acid transporters (LATs). Tryptophan hydroxylase (TPH) converts tryptophan to 5-hydroxytryptophan (5-HTP), which is quickly converted to serotonin (5-HT) by aromatic L-amino acid decarboxylase (AADC). TPH1 (body) and TPH2 (brain) require stabilization by 14-3-3 isoforms, including 14-3-3ε [26]. 5-HT is converted to *N*-acetylserotonin (NAS) by aralkylamine *N*-acetyltransferase (AANAT), which requires the presence of acetyl-coenzyme A (acetyl-CoA) and 14-3-3 isoforms, including 14-3-3ζ. NAS is converted to melatonin by acetylserotonin methyltransferase (ASMT). The acetyl-CoA requirement of AANAT intimately links the initiation of the melatonergic pathway with mitochondrial function, given that acetyl-CoA is primarily derived from the conversion of pyruvate to acetyl-CoA by the pyruvate dehydrogenase complex (PDC). The conversion of pyruvate to acetyl-CoA determines ATP production by the tricarboxylic acid (TCA) cycle and oxidative phosphorylation (OXPHOS), concurrently attenuating mitochondrial ROS production, thereby optimizing mitochondrial function.

**Figure 1:**
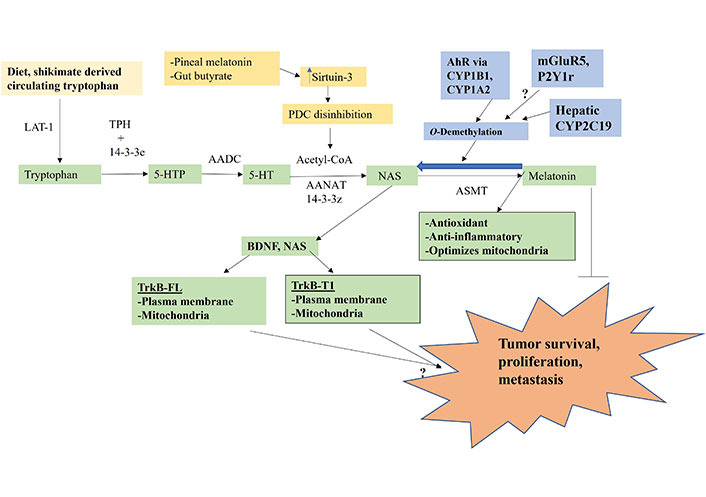
Tryptophan-melatonin pathway (green shade) can be intimately regulated by an array of systemic factors, including gut-derived tryptophan and butyrate and circadian-linked pineal melatonin, which concurrently regulate mitochondrial function as well as the melatonergic pathway. Factors influencing the NAS/melatonin ratio, especially AhR activation, will have significant impacts on tumor survival, proliferation and metastasis. P2Y1r: P2Y1 receptor; arrow (→): induces; T-bar (---|): suppresses; ?: requires data

Factors, including intercellular processes, modulating tryptophan, TPH, 14-3-3 isoforms, AANAT, acetyl-CoA, and ASMT will therefore determine the availability of the mitochondrial melatonergic pathway. Pro-inflammatory cytokine and stress/cortisol-induction of indoleamine 2,3-dioxygenase (IDO) and tryptophan 2,3-dioxygenase (TDO) lead to tryptophan being converted to kynurenine and kynurenine pathway products, such as kynurenic acid and quinolinic acid, which depletes tryptophan availability concurrent to altering wider physiology. IDO and TDO conversion of tryptophan to kynurenine activates the aryl hydrocarbon receptor (AhR), which “backward” converts melatonin to NAS via *O*-demethylation driven by AhR-induced cytochrome P450 enzyme 1A2 (CYP1A2), and CYP1B1 [27] as well as by hepatic CYP2A19 and the many factors and alleles that regulate hepatic CYP2C19 expression and function [28]. The *O*-demethylation of melatonin to NAS is of some importance in the tumor microenvironment as tumor cell exposure to melatonin invariably leads to tumor apoptosis [29], whilst NAS exposure may enhance the survival and proliferations of cancer stem-like cells via the capacity of NAS to mimic brain-derived neurotrophic factor (BDNF) by activating the BDNF receptor, tyrosine receptor kinase B (TrkB) [30]. This NAS/melatonin ratio is important as NAS not only activates the BDNF receptor, TrkB, but can induce BDNF resulting in BDNF and/or NAS activating the TrkB-full length (TrkB-FL) and TrkB-truncated-1 (TrkB-T1), both of which may be present on the mitochondrial and/or plasma membranes. Glutamate at the metabotropic glutamate receptor 5 (mGluR5) and purinergic 2Y1 (P2Y1) receptor activation may also “backward” convert melatonin to NAS to increase the NAS/melatonin ratio [3, 5] (Figure 1). The tumor regulation of the NAS/melatonin efflux from the variety of cells comprising the tumor microenvironment may therefore be a major determinant of tumor cell survival. Acetyl-CoA availability is crucial to the initiation of the melatonergic pathway and is regulated by a number of local and systemic processes, including pineal gland-derived melatonin and the gut microbiome-derived short-chain fatty acid, butyrate [31, 32]. Both pineal melatonin and butyrate increase the mitochondria-located sirtuin-3, which deacetylates and disinhibits PDC to enhance acetyl-CoA availability and therefore melatonergic pathway induction, as well as optimizing the mitochondrial TCA cycle and OXPHOS (Figure 1) [31].

Many of the systemic and local factors that regulate the interactions of the tumor microenvironment and tumor survival-proliferation-metastasis may be intimately linked to the mitochondrial melatonergic pathway and mitochondrial function. Tumor regulating receptors, such as the alpha 7 nicotinic acetylcholine receptor (α7nAChR), which melatonin induces [33], and the AhR, which significantly determines the NAS/melatonin ratio [34], as well as the TrkB-FL and TrkB-T1, upon which NAS induces proliferative effects via TrkB-FL [30] and mixed effects via TrkB-T1 [35], are all expressed on mitochondria and, as indicated, intimately interact with the melatonergic pathway. Consequently, the mitochondrial melatonergic pathway is closely associated with mitochondrial function and factors acting to regulate mitochondrial function, such as pineal melatonin and gut microbiome-derived butyrate. Importantly, the mitochondrial melatonergic pathway seems evident in all tumor microenvironment cells, with intercellular processes regulating the mitochondrial melatonergic pathway being important determinants of the function, fluxes, and dynamic interactions of all tumor microenvironment cells [36].

## Aging, cancer susceptibility, pineal melatonin, and cortisol

Aging is a major susceptibility factor for most medical conditions, including most human cancers [37]. The association of aging with the susceptibility to cancer and other medical conditions is classically linked to the raised levels of oxidative stress, leading to ROS-driven DNA damage and alterations in miRNAs that dysregulate patterned gene induction [38]. The raised levels of oxidative stress and inflammation over the course of aging that increase cancer risk are importantly determined by the dramatic, but gradual, ten-fold decrease in pineal gland melatonin production between childhood and the ninth decade of life [39]. The loss of the antioxidant, anti-inflammatory, and mitochondria optimizing effects of pineal melatonin at night are proposed to be an intimate aspect of cancer pathoetiology [40, 41]. Pineal melatonin increases sirtuin-3, which deacetylase and disinhibits PDC to increase acetyl-CoA availability for the initiation of the mitochondrial melatonergic pathway, concurrent to optimizing the TCA cycle and OXPHOS, thereby dampening any low level immune-inflammatory activity at night to reset cell function, including immune system cells, for the following day.

The aging-linked decrease in pineal melatonin, partly via the attenuation of melatonin’s upregulation of BAG-1 [42], will also determine the consequences of cortisol’s activation of the GR. Recent data shows BAG-1 to chaperone the GR to mitochondria and away from the nucleus, thereby significantly impacting GR driven patterned gene induction [43]. Melatonin also suppresses GR nuclear translocation by maintaining the GR in a cytoplasmic complex with heat shock protein 90 (Hsp90), as shown in peripheral blood mononuclear cells, where melatonin increases nuclear factor erythroid 2-related factor 2 (Nrf2)/heme oxygenase-1 (HO-1) as well as Bcl-2 and Bcl-2/Bcl-2-associated X (Bax) ratio to attenuate GR activation induced apoptotic susceptibility and immune cell function [44]. This has implications as to how the CAR and the stress-associated hypothalamus-pituitary-adrenal (HPA) axis activation can modulate the immune response and highlights the attenuation of GR effects by melatonin. The priming effect of pineal melatonin on GR translocation and effects has significant implications for the physiological consequences of CAR and during stress activation of the HPA axis [42], and therefore in how CAR and stress driven HPA axis activation associate with cancer pathogenesis [45], progression [46] and treatment [47]. This would indicate that pineal melatonin suppression over aging, as well as by inflammation [48], lipopolysaccharide (LPS) [49] and miRNAs [50] will have significant consequences for how CAR prepares systemic cells, including patterned immune responses, for the coming day.

**Figure 2:**
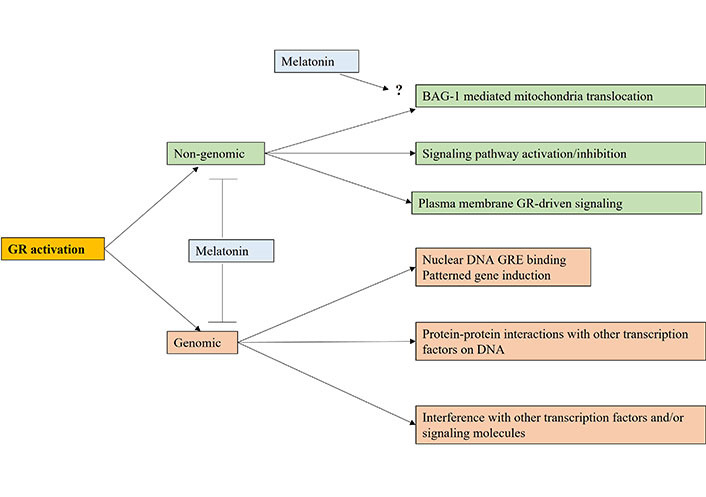
The variety of ways that cortisol activation of the GR can impact cell function via genomic and/or non-genomic mechanisms. Melatonin attenuates genomic and non-genomic effects by keeping the GR in a cytoplasmic complex with Hsp90 as well as epigenetically and perhaps directly upregulating BAG-1. Arrow (→): induces; T-bar (---|): suppresses; ?: requires data

Cortisol activation of the GR is an important aspect of tumor pathoetiology and pathophysiology, with effects primarily via the glucocorticoid response element (GRE) in gene promotors, thereby directly influencing patterned gene induction in the nucleus [51]. However, GR activation can influence immune function by a number of means [52], which require investigation as to how variations in pineal melatonin regulates diverse GR-driven processes (see Figure 2). The aging-linked decrease in melatonin and BAG-1 enhances GR nuclear translocation, with consequences not only for cancer pathogenesis but also for the nature of patterned immune responses, and intercellular interactions within a given microenvironment, including the tumor microenvironment. This is partly mediated by the GR activating the GRE in TDO [53], thereby depleting tryptophan for 5-HT and melatonin production, whilst increasing kynurenine, which activates the AhR to drive the CYP1B1 and CYP1A2 *O*-demethylation of melatonin to NAS, thereby increasing the NAS/melatonin ratio, with differential consequences for cell survival and proliferation, as well as for mitochondrial function and intercellular fluxes and interactions. The melatonin/Hsp90/BAG-1 suppression of GR nuclear translocation therefore impacts patterned gene transcription and cell plasticity responses by altering the mitochondrial melatonergic pathway, especially the NAS/melatonin ratio. The suppression of pineal melatonin over aging and in conditions associated with heightened cancer risk is therefore intimately linked to processes long associated with cancer pathogenesis and pathophysiology, such as GR nuclear translocation and TDO induction. The dramatic decrease in pineal melatonin therefore allows the CAR-driven GR to more readily translocate to the nucleus to upregulate TDO, kynurenine activation of the AhR and thereby increase the NAS/melatonin ratio. As tumor kynurenine is released to activate the AhR on natural killer (NK) cells and CD8^+^ T cells to induce “exhaustion” [54], the circadian interactions of pineal melatonin, BAG-1, CAR, and the AhR will significantly impact intercellular interactions in the tumor microenvironment to favor tumor survival when pineal melatonin is suppressed [8, 55].

**Figure 3:**
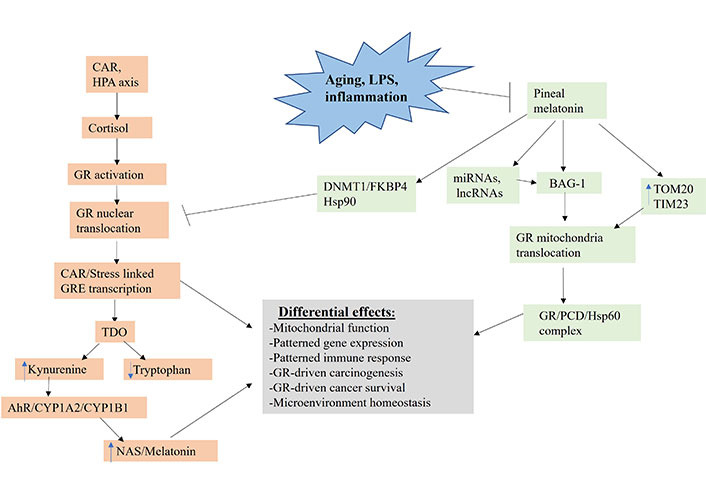
Shows how aging, LPS and inflammation, by suppressing pineal melatonin production, modulate how CAR, and stress HPA axis (orange shade) regulate the patterning of systemic gene expression and mitochondrial function. TIM23: mitochondrial import inner membrane translocase subunit 23; DNMT1: DNA methyltransferase 1; FKBP4: FKBP prolyl isomerase 4; lncRNA: long non-coding RNA; TOM20: mitochondrial import outer receptor subunit 20; arrow (→): induces; T-bar (---|): suppresses

This has significant implications for conceptualizing how circadian processes link to a wide array of aging-associated conditions, including cancer pathogenesis and prevention as well as intercellular interactions in the tumor microenvironment (Figure 3) [56].

GR activation, when translocated to the nucleus, primarily induces stress-linked genes expressing the GRE in their promotor, including TDO (Figure 3). TDO converts tryptophan to kynurenine, thereby increasing AhR activation, leading to the *O*-demethylation of melatonin to NAS. Tumor kynurenine release suppresses NK cells and CD8^+^ T cells, interacting with the effects of heightened GR activation in these and other tumor microenvironment cells. Pineal melatonin’s direct and indirect regulation, via miRNAs and lncRNAs, of BAG-1 (green shading), during the course of sleep prevents CAR induced GR nuclear translocation by translocating the GR to mitochondria, where it can have diverse effects. In the absence of melatonin induction of BAG-1, melatonin maintains the GR in a cytoplasmic complex with Hsp90. Melatonin also increases TOM20 and TIM23, which enhances GR translocation into the mitochondrial matrix, where it can form a complex with melatonin induced Hsp60 and PDC, thereby directly modulating mitochondrial function and metabolism. Pineal melatonin also suppresses GR nuclear translocation and effects via DNMT1/FKBP4. The dramatic suppression of pineal melatonin by aging, LPS, and pro-inflammatory cytokines therefore attenuates BAG-1 upregulation and, by a number of mechanisms, increases GR nuclear translocation, thereby altering the consequences of CAR and stress-linked HPA axis activation. This has significant impacts on how CAR and HPA axis activation increase cancer pathogenesis and pathophysiology via differential impacts on cancer cells, immune cells and other tumor microenvironment cells, thereby altering the dynamic homeostatic interactions of cells in the tumor microenvironment. The heightened levels of pro-inflammatory cytokines in the tumor microenvironment will also increase IDO (not shown in Figure 3 for clarity), which has similar kynurenine/AhR consequences as GR/GRE induced TDO.

Importantly, the different cells of the immune system have long been recognized to be differentially regulated by the circadian rhythm [57]. A plethora of data show melatonin [58, 59], including indirectly [60, 61] to modulate all immune cells, although seldom are the interactions of melatonin and GR activation investigated in immune cells. In studies investigating GR and melatonin interactions, it is clear that melatonin significantly modulates GR effects, including via FKBP4 regulation [62], as shown in immune cells [45]. It is widely recognized that glucocorticoid treatments, by suppressing NK cells and cytolytic cells, enhances cancer pathogenesis [61], thereby linking to how stress induced HPA axis activation and glucocorticoid treatment increase cancer susceptibility and modulate progression [63]. Glucocorticoids may also modulate the patterned immune response via the glucocorticoid induced tumor necrosis factor receptor (GITR) [64], which also induces IDO [65], being another route whereby CAR and HPA axis activation can suppress tryptophan and the tryptophan-melatonin pathway, whilst increasing kynurenine-driven AhR activation to heighten the NAS/melatonin ratio, to modulate cancers and their interactions with immune cells in the tumor microenvironment.

GR activation also acts via the induction of serum- and glucocorticoid-induced kinase-1 (SGK1) upregulation, which is a significant contributor to many cancers, including triple-negative breast cancer (TNBC), where it contributes to GR potentiation of metastasis and proliferation [66]. SGK1 is variably regulated by numerous transcription factors and epigenetic mechanisms [67], being a significant contributor to the circadian effects of CAR, as shown in spinal astrocytes [68]. SGK1 upregulates the T helper type 17 (Th17)/regulatory T cell (Treg) ratio by differentially modulating the development and function of both cells, thereby altering the nature of the patterned immune response and increasing “autoimmune”-linked processes [69]. The chronic stress/depression-mediated disruption of GR-driven negative feedback on the HPA axis is mediated via SGK1 activation, as shown in astrocytes [70]. SGK1 normally regulates ion homeostasis, promotes survival under stress and optimizes mitochondrial function [71], with SGK1 being protected against endoplasmic reticulum degradation by being maintained in the cytoplasm by association with lipid droplets and Hsp90 [72]. Such data indicates that calorie intake may be a significant determinant of SGK1 levels and effects, with some association as to the pathogenesis of obesity, metabolic syndrome and hypertension [73], and therefore in how metabolic dysregulation may associate with cancer risk [74]. Interestingly, chronic dexamethasone suppresses SGK1 in the hypothalamic arcuate nucleus, thereby increasing obesity and metabolic syndrome [75], indicating a significant GR effect in the hypothalamus that may be modulated by variations in pineal and local melatonin. SGK1 also contributes to cell survival by upregulating BAG-4 [76]. Whether SGK1 modulates other BAGs, especially BAG-1 and BAG-3 in different cell types will be important to determine. Overall, the GR can act via a number of processes to modulate cell function, with most data indicating primary effects via promotor GRE, and therefore on patterned gene induction. The differential effects of GR activation and melatonin in tumor microenvironment cells are looked at next.

## Cortisol, melatonin, TrkB and tumor microenvironment cells

Glucocorticoids are extensively used clinically, including in the regulation of allergies, rheumatic disorders, autoimmune conditions and when uncontrolled chronic inflammation is evident, as in the management of severe acute respiratory syndrome coronavirus 2 (SARS-CoV-2) in the coronavirus disease 2019 (COVID-19) pandemic where adjunctive melatonin may have proved more useful [61, 77]. The popularity of glucocorticoid treatments is based on their general anti-inflammatory effects and lymphocyte apoptosis [61], with glucocorticoid treatments generally acting to increase cortisol via the induction of local, cellular 11β-hydroxysteroid dehydrogenase type 1 (11β-HSD1) [78]. Detailed data in rodents shows the GR suppresses macrophages, neutrophils, mast cells, dendritic cells (DCs) [61, 79, 80] as well as the cytotoxic immune responses of NK cells, CD8^+^ T cells and Th1 cells, partly via the downregulation of interferon γ (IFN-γ) [61]. GR activation may also increase Th2 and Th17 immune cell responses, which contributes to a prolonged “mixed” inflammatory response, as well as increasing the activity and levels of B-cells immunoglobulins [81, 82]. The CAR is an important determinant of both the response and distribution of T cells over the circadian rhythm [61]. As indicated above, this data, especially *in vivo* CAR data, may be confounded by alterations in pineal and local melatonin, NAS/melatonin ratio and BAG-1 regulation. Importantly, GR activation enhances the pathogenesis and pathophysiology of cancer cells being generally linked to stress-induced HPA axis activation, in stress-linked cancer pathogenesis [61, 63]. As indicated, chronic stress modulates CAR, which is mediated via GR-induced SGK1 [70], leading to CAR being generally slightly suppressed. Suppressed CAR correlates with concurrent symptomatology in cancer patients, including depression and symptom burden [83]. Overall, chronic stress effects can modulate CAR and contribute to cancer pathogenesis, in association with wider symptomatology, with important effects on patterned immune cell responses.

Melatonin has contrasting effects to the GR on immune cells, generally suppressing the “immediate” responders, such as neutrophils, mast cells and macrophages [58, 84, 85], whilst enhancing the efficacy of the “second phase” responders, such as NK cells and CD8^+^ T cells [86, 87]. In tumor-associated immune cells, it is often the effects of melatonin on cancer cells that leads to altered fluxes and interactions within and among tumor microenvironment immune cells, such as neutrophils and CD8^+^ T cells [60, 88], given the powerful role of cancer cells in determining the phenotype and fluxes of other cells in the tumor microenvironment [8]. Melatonin’s effects on immune cells highlight the importance to tumor survival of limiting melatonin availability in the tumor microenvironment, as well as indicating the relevance of dynamic intercellular interactions in shaping the function of cells in the tumor microenvironment. Melatonin is often described as a homeostatic regulator due to its long evolutionary association with mitochondrial function [25], with melatonin effects partly dependent on the nature of the dynamic metabolic interactions in a given microenvironment and the intracellular metabolic function of a given cell. For example, melatonin increases mitophagy and PTEN-associated kinase 1 (PINK1)/parkin to suppress CD8^+^ T cell chemoattraction in “immune-mediated” conditions, such as Parkinson’s disease [2, 89], but suppresses mitophagy in cancer cells [90], whilst also derepressing CD8^+^ T cells [60]. However, melatonin generally has suppressive effects on pro-inflammatory responses by Th1 and Th17 cells, whilst potentiating Treg [91], in contrast to GR effects [81, 82]. Importantly, the direct optimizing effects of melatonin on NK cells and CD8^+^ T cells [86, 87], as well as indirect effects via altering tumor fluxes to cytolytic cells [60] are in sharp contrast to GR effects, as are melatonin effects in cancer cells.

TrkB is a significant suppression target in many cancer cells [92–95], as in many other conditions driven by excessive proliferation, such as endometriosis [96]. TrkB activation enhances the survival and proliferation of cancer stem-like cells as well as increasing metastasis, epithelial-mesenchymal transition (EMT), vascular invasion and poor prognosis [97–100]. As highlighted above (see Figure 1), TrkB effects are complicated by the presence of TrkB-FL and TrkB-T1 on the mitochondria and/or plasma membranes, with any differential effects of TrkB isoforms and membrane site still to be determined in tumor microenvironment cells. However, the capacity to induce NAS and NAS-induced BDNF clearly contributes to plasticity of cellular and mitochondrial function, including as driven by intercellular processes. NAS can also be metabolized to *N*-(2-(5-hydroxy-1*H*-indol-3-yl) ethyl)-2-oxopiperidine-3-carboxamide (HIOC), which can also activate TrkB [101]. The relevance of HIOC in cells of the tumor microenvironment is still to be determined.

The contrasting effects of melatonin, GR activation and TrkB activation on immune cells and cancer cells are indicated in Table 1. As is evident in Table 1, there is a paucity of basic data on the effects of TrkB in different immune cells, and an absence of any data on any differential effects arising from TrkB-FL and TrkB-T1 on the plasma membrane, *versus* mitochondrial membrane. The absence of such data considerably complicates interpretation of the consequences of changes in the data reviewed in Table 1.

**Table 1:** The differential effects of melatonin, TrkB and GR on immune cell pro-inflammatory activation, as well as in tumor cell survival/proliferation

**Cell type**		**Melatonin**	**TrkB**	**GR**	**References**
Immune cell	Macrophage	–	–	–	[84, 102, 103]
	Microglia	–	+	–	[23, 104, 105]
	NK cell	+	–	–	[106–108]
	CD8^+^ T cell	+	+	–	[56, 109, 110]
	DC	–	+	–	[111–113]
	Treg	+	?	+	[91, 114]
	Mast cell	–	?	–	[58, 80]
	Neutrophil	–	–/?	–	[52, 85, 115]
	Astrocyte	–	–/+	+	[116–118]
	MDSC	–	N/A	+	[59, 119]
Tumor cell		–	+	+	[17, 18, 93]

+: stimulatory; –: inhibitory; N/A: no data available; MDSC: myeloid-derived suppressor cell; ?: requires data. –/? is meant to represent some data suggestive of “inhibitory” but requires more data

TrkB effects are likely to be complicated by the presence of TrkB-FL and/or TrkB-T1 on the plasma membrane and/or mitochondrial membrane, thereby complicating the consequences of the NAS/melatonin ratio within individual cells as well as the dynamic intercellular interactions within the tumor microenvironment. The interactions of GR activation following CAR and stress-driven HPA axis activation are not only modulated by melatonin and BAG-1, as GR activation has a biphasic effect on TrkB levels, as shown in astrocytes [120]. These authors showed acute GR activation to increase phosphorylated TrkB at 1 h in association with increased BDNF induction, but with both TrkB and BDNF suppressed at subsequent timepoints over the next 24 h [120]. Such data would indicate that CAR and stress driven HPA axis activation of the GR will have distinct effects that are dependent upon variations in the pineal and local NAS/melatonin ratio. This requires investigation across body cells, including within the tumor microenvironment.

## Hypothalamic interactions of melatonin, GR, and TrkB in tumor microenvironment regulation

Notably, pineal melatonin has heightened and prolonged effects in the third ventricle, *versus* circulating concentrations [121]. This has particular consequences for hypothalamic function, including in the differential regulation of hormones associated with modulating feeding, metabolism, aggression and reproduction [42], with effects strongly determined via tanycytes that line the third ventricle and hypothalamic astrocytes [122]. Many hypothalamus regulated hormones, such as testosterone, estrogen, luteinizing hormone and oxytocin as well as hypothalamus-regulated pituitary hormones and thyroid hormones, also have differential effects on different immune cells, cancer cells and their interactions. The dramatic suppression of pineal melatonin over aging may therefore mediate some of its consequences, at least partly, via alterations in hypothalamic fluxes over the circadian rhythm, such as oxytocin.

Oxytocin is primarily produced in the supraoptic and paraventricular nuclei, with the stimulation of hypothalamic oxytocin neurons suppressing gastric cancer progression, as shown in mice [123]. This is proposed to be mediated via the induction of sympathetic neuronal activity in the celiac-superior mesenteric ganglion and consequent β2 adrenergic receptor activation [123]. However, it is also important to note that oxytocin is a partial agonist of the μ-opioid receptor [124] and regulator of the κ-opioid receptor [125], with the opioidergic system an important determinant of immune regulation across diverse medical conditions [126], including in the tumor microenvironment [127]. Overexpression of μ-opioid receptor messenger ribonucleic acid (mRNA) in 18 common solid cancers is predictive of poor prognosis [128]. Macrophage μ-opioid receptor activation suppresses macrophage phagocytosis of cancer cells [129], whilst μ-opioid receptor activation increases microglia reactivity [130]. The μ-opioid receptor is also a significant regulator of NK cells [131], CD8^+^ T cells [132] and tumor microenvironment DCs [133]. Such data indicate how oxytocin, via the opioidergic system, can alter the nature of the intercellular interactions in the pathogenesis and pathophysiology of the tumor microenvironment.

Pineal melatonin levels in the third ventricle not only modulate hypothalamic oxytocin [134], as melatonin also suppresses the κ-opioid receptor [135] and increases the endogenous μ-opioid receptor ligand, β-endorphin [136], indicating that the suppression of pineal (and possibly) local melatonin production will modulate the influence of the opioidergic system on patterned immune responses, including within the tumor microenvironment. The opioidergic system is also regulated by GR activation [137], indicating that melatonin and BAG-1 regulation of the GR will modulate the opioidergic system as well as wider physiological processes relevant to the intercellular interactions in the tumor microenvironment. The suppression of the opioidergic system over aging, including in the regulation of hypothalamic hormones [138], parallels the decrease in pineal melatonin over aging and, as indicated above, may be closely linked to melatonin’s regulation of the opioidergic system. Such data highlights the complexity of factors and their interactions that can regulate tumor microenvironment pathogenesis and progression, whilst also highlighting the diverse consequences arising from alterations in pineal melatonin production over the circadian rhythm. The reciprocal negative interactions of melatonin and the GR, including via BAG-1, also modulate hypothalamic fluxes with consequences for cancer pathoetiology and dynamic intercellular interactions in the tumor microenvironment. The interactions of oxytocin with the opioidergic system in the regulation of cancer pathophysiology may therefore be intimately linked to circadian interactions of pineal melatonin and CAR in modulating mitochondrial function and patterned gene transcription.

Clearly, melatonin-GR interactions will influence hypothalamic fluxes that may be differentially relevant in different types of cancer, such as gonadotrophin releasing hormone (GnRH) regulation of pituitary luteinizing hormone in the regulation of ovarian, breast and prostate cancers [139–141], with relevance as to how other types of ovarian conditions, such as polycystic ovary syndrome (PCOS) [142, 143] and endometriosis [144, 145], are associated with ovarian and breast cancer risk. Such data highlights the magnitude of factors that can influence intercellular interactions in the tumor microenvironment, whilst also indicating the importance of melatonin/BAG-1/CAR-GR interactions over the circadian rhythm in coordinating hypothalamic function with wider physiological processes across different organs and tissues.

This is further complicated by factors acting to increase the NAS/melatonin ratio, such as the AhR-induced CYP1A2 and CYP1B1 (Figure 1) [27, 28], which *O*-demethylate melatonin to NAS. NAS, by activating TrkB-FL and TrkB-T1 on the plasma membrane and/or mitochondrial membrane will have distinct effects in different immune cells, as well as indirect effects on immune and wider cells via the regulation of hypothalamic hormones/fluxes over the circadian rhythm. It is unknown as to whether NAS directly or indirectly modulates BAG-1 and GR site of translocation or whether it could do so differentially in different cell types/phenotypes via miRNA and/or lncRNA regulation. It is widely recognized that TrkB activation is a significant target in cancer treatment [92, 93], with TrkB activation driving the proliferation and survival of cancer stem-like cells [36, 146], with differential effects on immune cells, *versus* melatonin and GR, as indicated in Table 1. Such data strongly indicates that an increase in the pineal NAS/melatonin ratio at night would have direct systemic effects on tumor pathogenesis and pathophysiology as well as indirect effects via the circadian regulation by NAS at TrkB of hypothalamic hormones.

Pineal NAS/melatonin ratio effects and interactions in the hypothalamus also have systemic consequences. Alterations in the NAS/melatonin ratio, AhR levels and ligands, and hypothalamic/pituitary hormones, coupled with GR translocation to the nucleus, *versus* mitochondria, and GR-induced TDO will have differential effects in different microenvironment cells thereby altering the homeostatic interactions in different microenvironments across the body. It is such dysregulation of a given microenvironment’s homeostatic interactions that is proposed to underpin the emergence of autoimmune/immune-mediated conditions, such as type 1 diabetes mellitus (T1DM) [147] and Parkinson’s disease [148]. In such “immune-mediated” conditions, the homeostatic dysregulation seems powerfully determined by alterations in mitochondrial melatonergic pathway function, enhanced oxidative stress and the suppression of PINK1 driven mitophagy, coupled to the upregulation of major histocompatibility complex 1 (MHC-1) that chemoattracts CD8^+^ T cells to destroy pancreatic β-cells and substantia nigra pars compacta dopamine neurons, in the examples given [2]. Systemic processes therefore regulate the nature of local microenvironment homeostatic interactions, with differential effects on mitochondrial function in different cell types, including via intercellular processes regulating the tryptophan-melatonin pathway. For example, in T1DM, systemic processes modulate pancreatic islets local microenvironments, with alterations in local intercellular interactions then acting to suppress the mitochondrial melatonergic pathway in pancreatic β-cells [147]. Susceptibility genes for a given medical condition may therefore act on such intercellular microenvironment processes.

**Figure 4:**
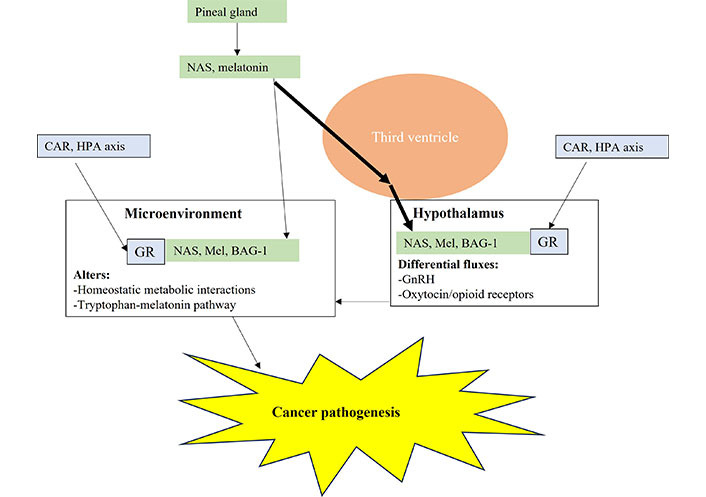
Pineal gland melatonin and NAS is directly released into the third ventricle where relatively heightened and prolonged levels are maintained over the night, *versus* circulating melatonin. This has direct and indirect, including hypothalamic, consequences for tumor microenvironment pathogenesis and ongoing pathophysiology. Mel: melatonin; arrow (→): induces

The circadian dysregulation highlighted above as arising from changes in the interactions of melatonin/BAG-1/GR act both directly and indirectly (e.g., via hypothalamic fluxes) to modulate the interactions in a given microenvironment. Cancer pathogenesis may therefore have overlaps with the processes that drive autoimmune/immune-mediated conditions via impacts on the intercellular homeostatic interactions in a given microenvironment (Figure 4). The emergent dyshomeostasis may drive cell elimination (“immune-mediated” disorders, such as T1DM and Parkinson’s disease) or wider control of the microenvironment by a given cell (tumor). Both these outcomes, “autoimmune” and tumorigenesis, may be importantly determined by mitochondrial melatonergic pathway suppression, such as in pancreatic β-cells in T1DM, and in different immune cells (NK cells, macrophages, CD8^+^ T cells) and/or by increasing AhR/CYP1A2/CYP1B1 and the NAS/melatonin ratio, in cells of the developing tumor microenvironment. This places cancer pathogenesis in a context of altered microenvironment homeostasis powerfully driven by systemic processes, leading to a given cell type(s) interacting with another cell to regulate the tryptophan-melatonin pathway, perhaps especially the NAS/melatonin ratio, in the course of dynamic alterations in homeostatic interactions.

The suppression of the local melatonergic pathway by streptozotocin [149] is proposed to underpin the loss of pancreatic β-cells in T1DM models [147]. The interactions of tumor microenvironment cells may overlap with this, via cancer cells regulating the tryptophan-melatonin pathway in other cells of the tumor microenvironment to optimize tumor survival and a tumor-led new pattern of homeostatic dynamic intercellular interactions in the ever-changing tumor microenvironment. This is exemplified by systemic cortisol/GR inducing tumor TDO and kynurenine release to activate the AhR on NK cells and CD8^+^ T cells, thereby driving cytolytic cell “exhaustion” [54], whilst altering the dynamic metabolic interactions and behaviors of tumor microenvironment cells. Whether AhR activation, via CYP1B1 and CYP1A2, in NK cells and CD8^+^ T cells, increases the NAS/melatonin ratio and NAS release to activate TrkB on cancer stem-like cells, thereby not only increasing survival and proliferation but also further changing the metabolic interactions in the tumor microenvironment will be important to determine. Such emphasis on the under-investigated tryptophan-melatonin pathway in tumor microenvironment cells is supported by data indicating the importance of the mitochondrial melatonergic pathway to the long-standing and continuing evolution of multicellular life on Earth [25], as well as the presence of mitochondrial receptors intimately associated with the mitochondrial melatonergic pathway, such as the AhR, TrkB and α7nAChR. These processes will be evident in the hypothalamus, and therefore to how the hypothalamus modulates wider systemic processes regulating tumor pathogenesis and tumor microenvironment homeostatic interactions.

As well as NAS and melatonin, GR activation is also a significant regulator of hypothalamic function and fluxes, including corticotropin-releasing hormone (CRH) in the initiation of the HPA axis. Pineal melatonin and NAS will therefore interact with BAG-1 to modulate hypothalamic GR effects, including in the regulation of oxytocin and GnRH (and consequently pituitary luteinizing hormone and gonadal hormones). Alterations in hypothalamic fluxes will interact with the direct effects of NAS/melatonin/BAG-1/GR in local microenvironment across the body to modulate the nature of the intercellular homeostatic interactions in a given microenvironment, including resident and infiltrating immune cells. Any emergent dyshomeostasis involves the dysregulation of the tryptophan-melatonin pathway by the emergent tumor cell of other cells in the tumor microenvironment, thereby initiating tumor pathogenesis (Figure 4).

The above provides a framework for understanding the complexity of processes underpinning tumor emergence and how it may be powerfully regulated by circadian and systemic processes, such as pineal melatonin and CAR, which change the intercellular and metabolic interactions in a given microenvironment, both directly and via the hypothalamus. Other systemic processes closely associated with aging-linked medical conditions, such as cancer, may be linked to this, including the role of the gut microbiome and gut permeability.

## Gut microbiome, tumor and mitochondrial melatonergic pathway

As with most medical conditions, alterations in the gut microbiome and associated gut permeability are closely linked to cancer pathogenesis, pathophysiology, and treatment [150, 151]. Although it is clear that much has still to be investigated regarding how the gut microbiome interacts with host pathophysiological processes, the suppression of the gut microbiome short-chain fatty acid, butyrate, seems invariably evident and relevant [151]. Butyrate can regulate a number of physiological processes, including: (1) as an epigenetic regulator via pan histone deacetylase inhibition (HDACi); (2) via G-protein coupled receptor 41 (GPR41), GPR43, and GPR109; (3) the capacity of butyrate to upregulate sirtuin-3, acetyl-CoA and the mitochondrial melatonergic pathway, thereby allowing gut microbiome-derived butyrate to optimize mitochondrial function across body cells [152, 153].

Much of the data regarding the role of the gut microbiome/permeability in cancer has focussed on gastrointestinal cancer, where butyrate suppresses EMT [154] and cancer pathogenesis, as well as chemotherapy side-effects [155]. However, butyrate and the gut microbiome/permeability have relevant effects in a wide array of cancers, including breast cancer [156], glioblastoma [157, 158] and ovarian cancer [159]. The mode of butyrate efficacy is variably defined across studies, including via its capacity as an HDACi [160], especially as HDACi is a general target in cancer treatment [161], as well as via epigenetic regulation by GPR43 activation suppressing methyltransferase-like 3 (METTL3) [154] and cancer regulating miRNAs subsequent to HDACi [160]. However, butyrate also has direct effects on immune cells, indicating that its relevance in cancer pathogenesis, course and treatment may be significantly determined by its regulation of immune responses.

Butyrate regulation of immune cells includes NK cells, where butyrate can enhance quiescent/suppressed NK cell function in the tumor microenvironment [162] and limit excessive activation [163], possibly mediated via butyrate’s upregulation of the mitochondrial melatonergic pathway, and the homeostatic regulatory effects of melatonin [152]. It is the effects of butyrate on liver NK (Kupffer) cell mitochondrial function over development that determines the cytotoxic efficacy of liver resident Kupffer cells [164], indicating the developmental role of the gut microbiome in the epigenetic regulation of immune responses relevant to cancer risk. As well as NK cells, butyrate enhances the cytotoxicity of CD8^+^ T cells via HDACi [165], highlighting the important role that alterations in the gut microbiome may have on the intercellular homeostasis of the tumor microenvironment. Butyrate also reverses the tumor microenvironment macrophage phenotype [166] and DC phenotype [167], further indicating the role of gut microbiome-derived butyrate in the regulation of the interactions of the tumor microenvironment. MDSCs are problematic in the tumor microenvironment, given their suppression of anti-tumor immunity. Butyrate depletes MDSC levels in the tumor microenvironment, as part of a wider regulation of tumor microenvironment immune cells and their interactions [168, 169]. Butyrate also impacts hypothalamic function, limiting microglia activation and associated hypothalamic inflammation [170], as well as regulating many hypothalamic and hypothalamic-pituitary fluxes, with immune regulatory consequences [171].

Clearly, butyrate effects may be importantly regulated by the presence of the tryptophan-melatonin pathway, thereby allowing butyrate to increase the mitochondrial melatonergic pathway [152]. Butyrate upregulates sirtuin-3 to deacetylate and disinhibit PDC, thereby increasing acetyl-CoA, which is a necessary cosubstrate for the initiation of the mitochondrial melatonergic pathway. Butyrate effects across cell types may then be dependent upon the availability of the mitochondrial melatonergic pathway. The butyrate induction of the melatonergic pathway has the potential to be problematic, under conditions of AhR activation that increases the NAS/melatonin ratio, giving the potential for released NAS to have trophic effects on tumor cells via TrkB activation. However, it is of note that NAS and AhR-induced CYP1B1 accumulation in the mitochondria of tumor cells induces apoptosis [172], indicating that the suppression of NAS mitochondrial efflux may be another important modulator of mitochondrial melatonergic pathway effects in tumor cells. This data would also indicate that AhR-induced CYP1B1 in mitochondria may be an important treatment target when coupled to mitochondrial melatonergic pathway activation in tumor cells. The different modes of butyrate effects (HDACi, GPR41, GPR43, GPR109, sirtuin-3) on the dynamic interactions of tumor microenvironment cells complicates its predictable effects in a given microenvironment, in the absence of relevant data.

Although melatonin is very highly produced in the gut especially after feeding, some pineal and exogenous melatonin effects are mediated via the gut microbiome, where melatonin upregulates butyrate-producing bacteria [173]. The suppression of pineal melatonin over aging will therefore have an impact on the gut microbiome, including levels of butyrate-producing bacteria [174], whilst the decrease in butyrate production will attenuate butyrate’s induction of the mitochondrial melatonergic pathway and optimization of mitochondrial function. Although the gut microbiome is an integral aspect of the circadian rhythm, the detailed nature of the processes underpinning this are still to be determined [175]. The interactions of melatonin (pineal and local) with butyrate and the gut microbiome may be an indirect route for the gut microbiome to regulate BAG-1 and therefore GR site of translocation.

**Figure 5:**
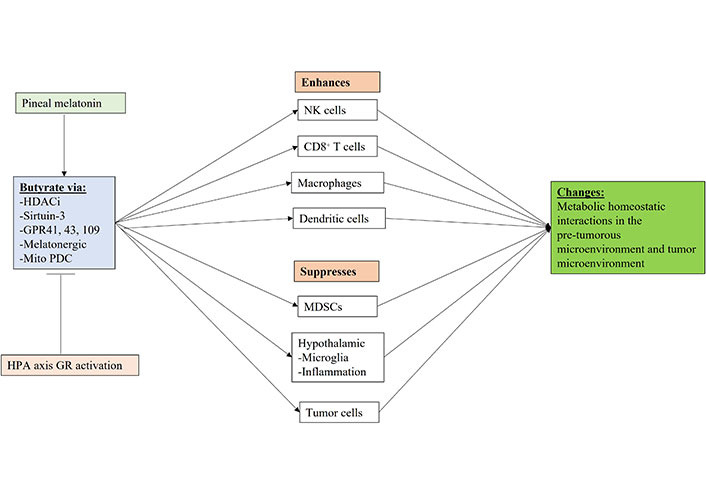
Shows how gut microbiome butyrate regulation, including by pineal melatonin and GR activation can regulate the function of immune cells and other cells in the pre-tumor microenvironment and tumor microenvironment. Mito: mitochondria; arrow (→): induces; T-bar (---|): suppresses

The gut microbiome also interacts with the GR, with GR activation in intestinal epithelial cells increasing gut permeability, endocytosis, and gut dysbiosis as well as systemic and hypothalamic inflammation, including via potentiating alcohol effects [176]. Such GR driven gut dysbiosis and increased gut permeability decreases butyrate production. The process of HPA axis activation initiated by CRH induction also increases gut permeability via CRH receptor 1 activation on mucosal mast cells, thereby increasing tumor necrosis factor α (TNFα) to increase gut permeability [177]. It has long been recognized that melatonin suppresses the gut permeability and dysbiosis induced by alcohol and stress, with melatonin effects mediated via the upregulation and activation of the α7nAChR, possibly driven by vagal nerve ACh efflux [178] onto enteric glial cells [3, 179]. Gut microbiome-derived metabolites of glycyrrhetinic acid-like factors (GALFs) inhibit 11β-HSD1 and11β-HSD2 and therefore the interconversion of cortisone and cortisol [180], indicating the significant role that the gut can have on local cortisol availability and GR activation. The gut microbiome is also a significant regulator of paraventricular hypothalamus CRH neurons, and therefore in the regulation of CAR and stress linked HPA axis activation [181]. Clearly, the gut microbiome and butyrate are in intimate two-way interactions with CAR/HPA axis/GR activation (Figure 5).

The regulatory effects of pineal melatonin and HPA axis/CAR driven GR activation on the gut microbiome derived butyrate are shown in Figure 5. Butyrate effects mediated by HDACi, sirtuin-3, GPR41, GPR43, GPR109, the melatonergic pathway and by the upregulation of mitochondrial PDC, thereby increasing ATP from OXPHOS and the TCA cycle as well as acetyl-CoA as a cosubstrate for the mitochondrial melatonergic pathway (Figure 5). The gut microbiome and butyrate production are regulated by many factors including diet, genetic and epigenetic as well as developmentally, which all act to modulate the interface of pineal melatonin and CAR/HPA axis driven GR activation within the circadian rhythm and the collective influence on metabolic interactions across microenvironments in cancer pathogenesis, as well as the established tumor microenvironment.

In an ILT-Mat tumor cell line and hepatoma cells, butyrate suppresses BAG-1 levels, thereby decreasing tumor survival [182, 183]. Whether this would indicate that the upregulation of endogenous mitochondrial melatonin in tumors suppresses the protection afforded by BAG-1 in tumors will be important to determine, including how this would impact the prosurvival effects of the GR in tumor cells. Data in retinoblastoma cells indicate that butyrate regulates the mitochondrial melatonergic pathway [184]. As to how butyrate modulates BAG-1 in non-neoplastic cells will be important to determine, including whether butyrate upregulation of melatonin allows butyrate to drive the derepression of miR-138 and wider epigenetic processes, thereby indirectly regulating BAG-1. It is not unlikely that many of the effects of butyrate, like melatonin, NAS, AhR and α7nAChR are mediated via direct effects on mitochondrial function and the consequences this has for wider coordinated cell activations.

Overall, the gut microbiome is an integral aspect of the circadian rhythm, with the capacity of butyrate to optimize mitochondrial function and upregulate the mitochondrial melatonergic pathway, indicating that the gut microbiome will be intimately linked to the interactions of melatonin/BAG-1/GR activation in tumors and other cells of the tumor microenvironment.

## Integrating melatonin, BAG-1, GR in tumor microenvironment

There is a growing appreciation of the role of the circadian rhythm in the pathogenesis and ongoing pathophysiology of cancer [40, 41]. Much of this work has centred on the role of pineal melatonin release at night and the heightened cancer susceptibility of factors and processes suppressing pineal melatonin, including aging, LPS, shift-work, T1DM, T2DM, increased LPS/gut permeability and raised levels of pro-inflammatory cytokines [48–50, 185]. The role of cortisol activation of the GR has predominantly been linked to stress-induced upregulation of the HPA axis given the roles of stress and GR activation in cancer cells, NK cells and CD8^+^ T cells in the tumor microenvironment [45–47]. CAR alterations have been primarily linked to stress-induced changes in hippocampal GR levels and altered negative feedback on CAR level and slope but with relatively little consequence for tumor pathophysiology.

It has been recognized since the 1980’s that pineal melatonin and CAR/HPA axis activation have some negative reciprocal interactions, with relevance across a diverse range of medical conditions [186]. The data reviewed above would indicate that pineal melatonin and CAR may be intimately linked aspects of the circadian rhythm, including by pineal melatonin’s direct and/or indirect upregulation of BAG-1 that will differentially regulate CAR systemic effects, including distinct effects in immune cells and in tumor microenvironments across the body. As well as possibly directly regulating BAG-1, pineal melatonin will indirectly increase BAG-1 via BAG-1 regulating miRNAs and lncRNAs, such as via the derepression of BAG-1 by the senescence-associated miR-138. BAG-1 upregulation, in association with melatonin keeping the GR in a cytoplasmic complex with Hsp90, decreases GR induced transcription and may increase GR translocation to mitochondria. Consequently, the interactions of pineal melatonin, BAG-1/Hsp90, and CAR/GR will have significant consequences for how body cells and systems are primed at the point of awakening. It is proposed that by having differential effects in the different cells comprising a given microenvironment, any suppression of pineal melatonin will alter any established homeostatic interactions in a given microenvironment, with implications not only for cancer pathogenesis but also for the pathogenesis of many “immune-mediated” conditions, such as T1DM and Parkinson’s disease.

Added complexity is provided by variations in the NAS/melatonin ratio, given the capacity of NAS to activate the BDNF receptor, TrkB [30]. This is further complicated by the expression of TrkB isoforms, TrkB-FL and TrkB-T1 in the plasma and/or mitochondrial membrane. The presence of other receptors linked to regulation of the melatonergic pathway on the mitochondrial membrane, including the AhR and α7nAChR, would indicate the importance of the mitochondrial melatonergic pathway to mitochondrial plasticity. As to how GR translocation to mitochondria modulates the mitochondrial melatonergic pathway and NAS/melatonin ratio, as well as mitochondrial membrane TrkB-FL, TrkB-T1, AhR and α7nAChR will be important to determine.

Systemic plasticity may also be regulated by factors modulating the pineal NAS/melatonin ratio, given the differential consequences of NAS, *versus* melatonin, in tumor cells and immune cells of the tumor microenvironment (Table 1), as well as in the hypothalamus. Pineal melatonin regulation of hypothalamic function will have significant consequences for how hypothalamic fluxes of oxytocin, opioids and reproductive hormones regulate the homeostatic interactions within microenvironments, including in the pathogenesis and pathophysiology of the tumor microenvironment.

The gut microbiome and gut permeability are also an integral aspect of the circadian rhythm, with gut microbiome-derived butyrate inducing and interacting with the melatonergic pathway in the course of optimizing mitochondrial metabolism, whilst GR activation is a significant determinant of gut permeability and gut dysbiosis. The gut microbiome is therefore intimately linked to both pineal melatonin and CAR/HPA axis activation, with consequences for butyrate levels and the butyrate optimization of mitochondrial function and therefore for cancer cell and immune cell function and interactions.

As to how wider data on tumor microenvironment pathophysiology can be linked to the above will be important to determine. For example, the retinoblastoma protein (Rb) is typically described as a tumor suppressor, with its level of phosphorylation and mutation being a significant predictor of cancer phenotype and treatment outcome [185]. Rb also interacts with BAG-1, where it seems to increase the translocation of BAG-1 to the nucleus [187]. This is given some support by long-standing data showing Rb to enhance GRE-driven transcription in the nucleus [188], with Rb proposed to be a relevant aspect of glucocorticoid sensitivity and negative feedback to the HPA axis [189]. As to whether an enhanced Rb/GR/BAG-1 nuclear translocation is relevant to the pathogenesis of cancers, e.g., via enhanced TDO upregulation, will be interesting to determine, including how circadian melatonin interacts with Rb. The nature of the Rb interaction with BAG-1 that changes its site of translocation to the nucleus will be important to determine, including whether Rb/BAG-1 nuclear translocation is what underpins Rb-mediated enhancement of GR effects at GRE. This data on Rb may overlap with recent work indicating that many classic “tumor suppressors” may be “double agents” by acting in an oncogenic manner, as perhaps exemplified by Rb nuclear interactions with BAG-1 [190]. It requires investigation whether the suppression of pineal and/or local melatonin’s capacity to keep the GR in a cytoplasmic complex with Hsp90 [44] contributes to enhanced Rb/GR/BAG-1 nuclear translocation in cancer pathogenesis, leading to TDO/kynurenine/AhR driven increases in the NAS/melatonin ratio in the precancerous microenvironment cells, thereby enhancing TrkB-driven proliferation, as an aspect of wider alterations in homeostatic, intercellular, metabolic interactions. This has a number of future research and treatment implications.

## Future research implications

The data highlighted above provide a framework for novel investigations in the pathoetiology and pathophysiology of cancer, as indicated below:

(1) Does pineal and/or local melatonin directly upregulate BAG-1?
(2) Does the indirect, epigenetic regulation of BAG-1 by melatonin, e.g., via the derepression of BAG-1 by miR-138 have relevance to the pathogenesis and/or pathophysiology of different cancer cell types and/or to the development of a pre-cancer microenvironment?
(3) Does pineal melatonin’s regulation of BAG-1 and the maintenance of the GR in a cytoplasmic complex with Hsp90 modulate the CAR, allowing the CAR to differentially prime the patterned immune response at the start of a given day? Does this provide a framework for conceptualizing how the circadian system modulates cancer pathogenesis and pathophysiology?
(4) How does the melatonin/BAG-1/GR interactions over sleep and awakening modulate individual immune cells, including macrophages, NK cells and CD8^+^ T cells, all of which show indication of circadian regulation. How do variations in the pineal NAS/melatonin ratio interact with CAR-driven GR activation to modulate the patterned immune response over the circadian rhythm?
(5) If indeed, the mitochondrial melatonergic pathway is a core feature of cell function, plasticity and intercellular interactions, does this provide a more realistic basis to link the diverse, and often disparate, bodies of data linked to cancer pathogenesis and pathophysiology, as indicated by the plethora of fluxes, miRNAs, lncRNAs and mutated proteins proposed to be important determinants of tumor pathophysiology. The investigation of the endogenous melatonergic pathway across most immune cells is prominent by its absence, with the exception of microglia and macrophages [23, 24], despite the dramatic effects of exogenous melatonin on immune cell function and phenotype.
(6) Is the potentiation of GR effects in cancer cells, as driven by heightened Rb/BAG-1 binding, important to cancer pathogenesis via heightened TDO induction, thereby enhancing proliferation via kynurenine activation of the AhR leading to an increase in the NAS/melatonin ratio in neighboring cells? Clearly, this is relevant to the induction of “exhaustion” in tumor microenvironment CD8^+^ T cells and NK cells [54]. However, would this also be relevant in tumor pathogenesis? For example, GR driven SGK1, seems to induce a cancer stem-like phenotype [66], suggesting a possible impact on tumor pathogenesis, in association with heightened TDO/kynurenine/AhR mediated rise in the NAS/melatonin ratio in microenvironment cells.
(7) How relevant is the potentiation of GR nuclear translocation by Rb and the nuclear Rb/BAG-1 complex in cancer pathogenesis? Does this have pathogenetic relevance mediated by enhanced GR induction of TDO and kynurenine efflux to neighboring cells to activate the AhR and increase the NAS/melatonin ratio? Does the suppression of endogenous melatonin alter the mitochondrial function of AhR-expressing neighboring cells, thereby changing the nature of the dynamic intercellular homeostatic interactions?
(8) As well as activating melatonin receptors (MTRs), MT1R and MT2R, pineal and local, exogenous melatonin may also be directly taken up into mitochondria by the peptide transporter 1/2 (PEPT1/2), whilst the sulphation metabolites of melatonin can be taken into mitochondria by the organic anion transporter 3 (OAT3) [191]. Factors acting to regulate PEPT1/2 and OAT3 provide other aspects of how melatonin regulates the plasticity of cellular and mitochondrial responses, such as the suppression of PEPT1/2 over aging [192]. How relevant is the direct uptake of pineal and local melatonin into mitochondria in the cells of the tumor microenvironment?
(9) Does the suppression of pineal and/or local melatonin’s capacity to keep the GR in a cytoplasmic complex with Hsp90 [44] contribute to enhanced Rb/GR/BAG-1 nuclear translocation in cancer pathogenesis, thereby enhancing TDO/kynurenine/AhR activation and NAS/melatonin ratio? Is this relevant in NK cells and CD8^+^ T cells suggesting that NK cells may be “inverse double agents” by providing trophic support to developing tumors via released NAS?
(10) Data shows astrocyte TrkB to be biphasically regulated over 24 h by GR activation [120]. How relevant is this in the tumor microenvironment as well as to variations in TrkB-FL *versus* TrkB-T1, and plasma membrane, *versus* mitochondrial membrane, TrkB expression in cells of the tumor microenvironment? How do variations in the NAS/melatonin ratio that may be present in the tumor microenvironment modulate GR effects.
(11) What mediates the mitochondrial translocation of the AhR, TrkB, and α7nAChR? Given the association of these receptors with the mitochondrial melatonergic pathway, do variations in the mitochondrial melatonergic pathway and/or mitochondrial oxidative status modulate the translocation of these receptors to mitochondria?
(12) How do BAG-1 regulating processes modulate other BAGs, such as BAG-2, BAG-3 and BAG-4? Is this coordinated by variations in the mitochondrial melatonergic pathway? BAG-3 may be particularly interesting to investigate given that BAG-3 physically interacts with signal transducer and activator of transcription 3 (STAT3) to stabilize STAT3 and endow some stem-like qualities in glioblastoma multiforme (GBM) cells [193]. Peroxisome proliferator-activated receptor γ (PPARγ) inhibition downregulates BAG-3 and 14-3-3γ, which inhibits the stem-like qualities in GBM [194]. BAG-3 ko decreases 14-3-3ζ [195], suggesting a concurrent attenuation of AANAT stabilization by 14-3-3ζ. BAG-3 ko also increases PINK1 and parkin [196], indicating mitophagy upregulation, concurrent to suppression of the mitochondrial melatonergic pathway. Would this be relevant to induction of stem-like qualities by disengaging the melatonergic pathway from mitophagy regulation as a core “phenotype” change that is less susceptible to “autoimmune” type cell elimination from neighboring cells driving suppression of the tryptophan-melatonin pathway?
(13) SGK1 upregulates BAG-4 [76]. Does SGK1 modulate other BAGs, indicating a role for CAR and HPA axis activation in BAG regulation in different cell types?
(14) Recent work indicates that mitochondria functions like a Tesla battery by being comprised of multiple independent “batteries” on the mitochondrial inner membrane, providing some variations for the levels of energy provided given that all “batteries” do not always fire simultaneously [197]. Does the GR modulate such “battery” capacity when translocated to mitochondria? Do other mitochondria located receptors linked to the melatonergic pathway, such as TrkB, AhR and α7nAChR modulate the capacity of “batteries” to be synchronized? Mitochondrial dynamics are powerfully regulated by the circadian clock, showing a metabolic oscillatory rhythm [198], indicating a significant role for pineal melatonin modulation of circadian genes as well as sirtuin-1 and sirtuin-3, which are intimately involved in regulating the metabolic circadian rhythm [198]. As to how CAR and the GR translocation to mitochondria interface with this will be important to determine, especially as disordered cristae on the inner mitochondrial membrane increase superoxide production [199].
(15) Importantly, the mitochondrial calcium uptake 1 (MICU1) not only interacts with the mitochondrial contact site and cristae organizing system (MICOS), but also optimizes MICOS regulation of cristae formation [200]. Melatonin increases MICU1 [201], indicating a significant role for variations in melatonin in core aspects of mitochondrial function and energy production as well as ROS production, with effects that will be attenuated with aging. MICU1 decreases with age in macrophages, thereby increasing nuclear factor kappa-light-chain-enhancer of activated B cells (NF-kB) driven macrophage inflammatory response [202]. How this interacts with lost pineal melatonin with age and possibly within macrophages and other cell types will be important to determine.

## Treatment implications

The frame of reference provided above not only integrates previously disparate data on tumor pathogenesis and pathophysiology but also provides a number of treatment targets:

(1) Treatments targeted to the tryptophan-melatonin pathway, especially the mitochondrial melatonergic pathway, in particular cells will shape treatments to core physiological processes regulating homeostatic interactions in the tumor microenvironment.
(2) Prevention may be targeted by interventions aimed at maintaining pineal melatonin production, especially over aging with relevance to a host of diverse medical conditions. Melatonin supplementation currently available only restores circulatory melatonin levels but does not replicate the maintained high melatonin levels in the third ventricle and the impact this has on core physiological processes regulated by the hypothalamus. Current melatonin supplementation also fails to take into account the plasticity afforded by variations in the pineal NAS/melatonin ratio, given that NAS released by the pineal gland at night is always evident.

## Conclusions

The capacity of pineal melatonin to upregulate BAG-1 and keep the GR in a cytoplasmic complex with Hsp90 has significant implications for how CAR primes the immune system for the upcoming day. This provides a novel conceptualization of relevant circadian processes in the course of sleep and awakening and provides an impetus for more imaginative investigations as to the physiological role of CAR in health and disease. This also provides a distinct perspective as to how variations in melatonin/BAG-1/GR can modulate the homeostatic interactions of the tumor microenvironment over the circadian rhythm. Given the powerful roles of melatonin and CAR in the regulation of patterned immune responses, the interactions of these two previously disparate aspects of the circadian rhythm provides a perspective on which broad bodies of data can be placed. As the dysregulation of the pineal melatonin/BAG-1/CAR/GR location has differential effects in different cell types, there are impacts on the intercellular homeostatic interactions occurring across previously established intercellular microenvironments. These intercellular homeostatic changes are primarily mediated by alterations in mitochondrial function and interaction across cell types, with the developing tumor microenvironment emerging from the capacity of tumor cells to regulate the mitochondrial melatonergic pathway in other microenvironment cells.

## Abbreviations

5-HT: serotonin
5-HTP: 5-hydroxytryptophan
AANAT: aralkylamine *N*-acetyltransferase
acetyl-CoA: acetyl-coenzyme A
AhR: aryl hydrocarbon receptor
ASMT: acetylserotonin methyltransferase
BAG-1: B cell lymphoma-2-associated athanogene 1
Bcl-2: B cell lymphoma-2
BDNF: brain-derived neurotrophic factor
CAR: cortisol awakening response
CRH: corticotropin-releasing hormone
CYP1A2: cytochrome P450 enzyme 1A2
DCs: dendritic cells
DNMT1: DNA methyltransferase 1
FKBP4: FKBP prolyl isomerase 4
GnRH: gonadotrophin releasing hormone
GR: glucocorticoid receptor
GRE: glucocorticoid response element
HPA: hypothalamus-pituitary-adrenal
Hsp90: heat shock protein 90
IDO: indoleamine 2,3-dioxygenase
lncRNA: long non-coding RNA
LPS: lipopolysaccharide
MDSC: myeloid-derived suppressor cell
MICU1: mitochondrial calcium uptake 1
miRNAs: microRNAs
NAS: *N*-acetylserotonin
NK: natural killer
OXPHOS: oxidative phosphorylation
PDC: pyruvate dehydrogenase complex
PEPT1/2: peptide transporter 1/2
PINK1: PTEN-associated kinase 1
Rb: retinoblastoma protein
ROS: reactive oxygen species
SGK1: serum- and glucocorticoid-induced kinase-1
T1DM: type 1 diabetes mellitus
TCA: tricarboxylic acid
TDO: tryptophan 2,3-dioxygenase
Th17: T helper type 17
TIM23: mitochondrial import inner membrane translocase subunit 23
TOM20: mitochondrial import outer receptor subunit 20
TPH: tryptophan hydroxylase
Treg: regulatory T cell
TrkB: tyrosine receptor kinase B
TrkB-FL: tyrosine receptor kinase B-full length
TrkB-T1: tyrosine receptor kinase B-truncated-1
α7nAChR: alpha 7 nicotinic acetylcholine receptor

## Declarations

### Copyright

© The Author(s) 2023.
